# On the Impact of Information Technologies Secondary-School Capacity in Business Development: Evidence From Smart Cities Around the World

**DOI:** 10.3389/fpsyg.2021.731443

**Published:** 2021-12-14

**Authors:** Virginia Barba-Sánchez, Luis Orozco-Barbosa, Enrique Arias-Antúnez

**Affiliations:** ^1^ENSITMA R.G., Department of Business Science, Universidad de Castilla-La Mancha, Albacete, Spain; ^2^I3A, Department of Computer Systems, Universidad de Castilla-La Mancha, Albacete, Spain

**Keywords:** Smart City, IT facilities, STEM, business activity, secondary education

## Abstract

Smart City initiatives across the globe have spurred increasing demand for high-skilled workers. The digital transformation, one of the main building blocks of the Smart City movement, is calling for a workforce prepared to develop novel business processes. Problem-solving, critical and analytical thinking are now the essential skills being looked at by employees. The development of the so-called STEM curriculum, Science, Technology, Engineering, and Mathematics is being given a lot of attention by educational boards in response to preparing young generations for the Smart City work market. Based on the IMD Smart City Index, PISA, and World Bank reports, we develop a model for assessing the impact of the IT secondary school capacities on Smart-City business developments. The model reveals the relationship between the technological capacity of the secondary-school, and the business activity of a Smart City. Moreover, the study shows the existence of a positive relationship between the IT capacity of secondary schools and the resulting entrepreneurial activity of the city. Our results are of interest to decision-makers and stakeholders responsible for designing educational policies and agents involved in the digital transformation and development of Smart Cities initiatives.

## Introduction

The Smart City concept (Giffinger et al., 2007; Hollands, 2008, p. 306; Kondepudi et al., 2014; Albino et al., 2015; Ben-Letaifa, 2015, p. 141; Richter et al., 2015, p. 214) has been attracting the attention of city authorities for more than a decade. Nowadays, nobody questions that Smart City is something more than a fashionable label (Kraus et al., 2015). In fact, Smart City initiatives around the world are seen as a major revolution in the sustainable development of urban areas (Kitchin, 2014; Hollands, 2015) providing a framework of governance aiming to manage resources in a more efficient way (Achaerrando et al., 2012) as well as facilitating an environment conducive to entrepreneurship (Richter et al., 2015). Most of the Smart City definitions have been summarized in Ramaprasad et al. (2017) and reviewed in Sánchez-Corcuera et al. (2019).

Information and communications technologies infrastructures and services are key to the development of Smart City (Samih, 2019) and more importantly to the creation of new business models by enabling the automation and optimization of processes as a driver of entrepreneurial and innovative ecosystem (Sauer, 2012; Bifulco et al., 2016; Barba-Sánchez et al., 2019; Florida et al., 2019). In fact, “Smart cities initiatives try to improve urban performance by using data, information and information technologies (IT) to provide more efficient services to citizens, to monitor and optimize existing infrastructure, to increase collaboration among different economic actors, and to encourage innovative business models in both the private and public sectors” (Marsal-Llacuna et al., 2015). Within this process, the role of education in the growth of economic activity as an accelerator of the process of technological diffusion and training of human capital is widely recognized (Nelson and Phelps, 1965; Akhvlediani and Cieślik, 2020). Acs et al. (2005) state that “… part of public sector expenditure devoted to education, and education has been shown to be positively associated with entrepreneurship” or “both expenditures on education and economic growth are positively related to entrepreneurship.” It is evident that education, being one of the key pillars of our society, plays a major role on the success of Smart City initiatives.

At the beginning of this century, the need for new education paradigms have been deemed necessary to keep abreast with technological developments and social/economic changes. This movement gave birth to the STEM education paradigm. This paradigm has as one of its main goals to provide students with the skills required to join the labor force in critical sectors of growth of the most advanced economies. Different to previous curriculum changes, the STEM curriculum goes a step ahead on the teaching/learning methodology and aims of simply learning the technical and scientific principles of technical core areas (International Technology Education Association (ITEA/ITEEA), 2009). STEM education initiatives are being tailored to contribute to the acquisition and development of problem-solving, critical thinking, and analytical thinking abilities and capacities. Students are guided to better lead real-world connections in the curriculum (Brown et al., 2011; Süheyla, 2019). STEM education includes activities motivating students’ interest and orientations toward science, technology, engineering, and mathematics fields while acquiring skills required in today’s workplaces.

The main contribution of this study is to provide a new approach to ascertain the relationship between education, with a focus on the IT education paradigm, and economic growth, also providing empirical evidence in a new context, the Smart City, and for non-university educational levels. Furthermore, other contributions have to do with citizens’ perception of the intelligence of the Smart Cities and how their satisfaction in this respect can also influence local economic growth; and with the role played by citizens’ prioritization of education in this economic growth. The paper therefore focuses on the foundational issue of secondary education quality and highlights its contribution on fully exploiting the productivity potential of urbanization. In effect, our study shows the benefits of concentrating investments on competitive cities rather than spreading it across all the periphery and the need of ensuring equality of opportunity for individuals to exploit their potential.

Different to most studies, this work investigates the impact of secondary education IT capacities, rather than tertiary or higher education, over the business activities in the context of Smart City initiatives. The rationale behind our choice is due to the increasing training and use of IT technologies in the early stages of education and more important, to the importance of spurring secondary students’ interest in tertiary STEM education. The paper takes as starting point the data of the report IMD Smart City Index (2019) by IMD World Competitiveness Center’s Smart City Observatory in partnership with Singapore University of Technology and Design. The report ranks 102 cities around the world and “uniquely focuses on how citizens perceive the scope and impact of efforts to make their cities smart, balancing economic and technological aspects with humane dimensions.”

The paper is structured as follows. Section “IT Education: A Key Element of Smart City Initiatives” introduces a general overview of IT education and its relevance on the development of the workforce in the context of the ongoing Smart City initiatives. Sections “IT Education: A Key Element of Smart City Initiatives, IT Secondary-School Capacity and Smart City, and Smart City and Business Activity” introduce the hypothesis of our model establishing the relationship between the deployment of IT secondary education services, and the level of satisfaction that the citizens have over the success of Smart City’s economic development. In particular, Section “IT Education: A Key Element of Smart City Initiatives” motivates the relationship between, IT school capacities and the resulting Smart City business activity. Section “IT Secondary-School Capacity and Smart City “then relates the Smart City and the resulting business activity. Section “Smart City and Business Activity” looks at the relationship between, education in general with emphasis on IT education capacities resulting in business activity. Section “IT Education and Business Activity” describes the sources of information used in the numerical evaluation of the proposed model. Section “Empirical Evaluation” presents the empirically results of the proposed model. Section “Main Findings and Discussion” summarizes and analyses the main findings derived from the model results. Section “Conclusion and Future Research” outlines the conclusion, shortcomings, and future research lines.

## Information Technologies Education: A Key Element of Smart City Initiatives

Recent works have shown the importance of STEM learning at secondary in attracting students to STEM fields in tertiary education (Chachashvili-Bolotin et al., 2016). We should notice that the studies being carried out on the evaluation of STEM curriculum have focused on the benefits or shortcoming of the increasing use of IT in schools (Hu et al., 2018; Xiao et al., 2019). The work presented herein goes a step beyond by taking into account not only the IT infrastructure put in place worldwide in secondary education institutions, but it also looks into the abilities of educators and students. In this context, the teaching staff requires to keep up with the latest IT developments and students have to play a more active role in the learning process. The ultimate goal of this work is to identify the relationship between the overall IT secondary education capacities and the resulting entrepreneurial activity of the ongoing Smart City initiatives; where the capacities comprise the IT infrastructure and teaching staff skills.

Hazelkorn et al. (2015) stated that “Science education should be an essential component of a learning continuum for all, from pre-school to active engaged citizenship” and “Science education should focus on competences with an emphasis on learning through science and shifting from STEM to STEAM by linking science with other subjects and disciplines.” As a result of the study conducted by Hazelkorn et al. (2015) the authors proposed the Framework for Science Education for Responsible Citizenship. This framework provides a comprehensive set of objectives, recommendations and actions. Among the set of recommendations, the framework states that STEM education should be complemented by introducing students into entrepreneurship. This vision has been shared by others. In a recent report by Johnson and Mansfield (2019) from Monash University, the authors state: “… entrepreneurial education has the power to transform. Adding it to STEM programs in schools provides students with crucial future-ready skills and an array of new career possibilities.” Furthermore, we should realize that STEM education has to be complemented by introducing students into entrepreneurship.

As evidence of the importance of IT in the early stages of education and the lack of workers in these areas, the Obama administration established the program Stem for All [S4A] (2016). Similar initiatives in Europe, e.g., European Commissioner for Education, Culture, Youth, and Sport stress the relevance of training young students in STEM areas starting at the early years of education (EU STEM Coalition, pp. 5). Notice that Obama Administration actions, and being continued under Trump administration, as well as those proposed by the EU STEM Coalition are in line with the seven criteria that characterize a smart partnership defined in the EDUsummit15 (Davis et al., 2015; Leahy et al., 2016).

Moreover, for the purpose of the work presented here, the following Sustainable Development Goals of the 2030 Agenda have especial relevance (2030 Agenda, 2015):

• Goal 4. Ensure inclusive and equitable quality education and promote lifelong learning opportunities for all.
• Goal 8. Promote sustained, inclusive and sustainable economic growth, full and productive employment and decent work for all.

Meeting the first goal should ensure the universal access to quality education throughout all the phases of education, including early stages. The second could be achieved in part throughout the education and training of high-educated individuals and entrepreneurship.

Taking into account the great interest and the number of resources being put into STEM education and IT capacities, the main question is:

*how much are the STEM Education curricula and IT infrastructures at Secondary School contributing to the success of ongoing Smart City initiatives, and more precisely to their economic wealth?*

This question is increasingly being brought due to the increasing role played by STEM in the economy of our countries. In words of Freeman et al. (2019) “We (also) need to understand better the links between STEM education, research, and the economic wealth of a country and wellbeing of its citizens.” One of the key criteria of this smart partnership characterization is “(to) enhance the quality of education *via* digital technologies (ICT)” (Davis et al., 2015: 10). In fact, according to Chai et al. (2019) “the teachers’ efficacies of integrating technology into science, mathematics and engineering subject predict their efficacy of integrative STEM teaching.” Similar ideas are described in Chen et al. (2019).

It is important to clarify that the worldwide adoption of STEM curriculum still in its infancy stages. Bearing this fact on mind and the fact that the implementation of STEM curriculum will highly rely on the IT capacities of the school, the main aim of this study is to develop a model revealing the relationship between the IT capacity of the secondary-school, and the business activity of a Smart City. Toward this end, the PISA report provides statistics related to the IT infrastructures and IT teaching staff skills. As already explained in the introduction, this information is complemented by other data sources.

## Information Technologies Secondary-School Capacity and Smart City

In recent years, a holistic vision of Smart City has emerged in the academic world, centered on the human being and balancing social, cultural, environmental, economic, and technological aspects (Mora et al., 2017), as a response to the techno-centric vision (Zanella et al., 2014). This latter vision has been largely criticized as being very focused on the underlying infrastructures used on the implementation of novel urban services (Hollands, 2008). By placing the focus on the citizen, recent studies have identified that education, learning, and knowledge are key elements to successful Smart City initiatives (Leahy et al., 2016). Moreover, smart partnerships must be inherent to Smart City development leading to Smart Citizens. A smart partnership must include different actors in education including teachers, researchers, organizations but also government, industry, civil society (Davis et al., 2015). That is to say, smart partnership for education should involve the main actors in charge of developing Smart City initiatives. In fact, one key element of a Smart City is Smart Education; and Smart Education should be seen as the backbone for successful and ever-growing development of Smart City initiatives.

Smart City developers should acquire the skills and knowledge to understand the overall scope of what it is involved in a Smart City including services requirements and the latest available technology. They should continuously update their STEM-knowledge base making use of the latest IT technologies allowing them to gain timely and ubiquitous access to high-quality educational contents. A good example of this trend is the Smarter Education initiative as a part of the global Smarter City initiative by IBM (Rudd et al., 2009; IBM, 2012). In fact, our work is aligned with Williamson’s vision (Williamson, 2015) which states that “education is being positioned as a laboratory space for the enactment of big data practices and as a social mechanism for the production of ‘smart citizens’ who can participate actively and productively in the big data dynamics of the smart city.”

This study undertakes the development of a model for evaluating the impact of IT secondary school ecosystems on the process of constructing the Smart City vision by considering the technological facilities and, more importantly, the teaching staff IT abilities. The model is initially constructed on the following hypotheses:

Hypothesis 1: The school IT capacity influences citizens’ satisfaction with the city’s infrastructure.

Hypothesis 2: The school IT capacity influences the adoption of IT by the city.

Hypothesis 3: The adoption of IT by the city mediates the relationship between the school IT capacity and the citizens’ satisfaction with the city’s infrastructure.

## Smart City and Business Activity

The conception of the Smart City as an ecosystem of new innovation opportunities for large and small regional companies, pointed out by Hollands (2008), is still valid (Lee et al., 2014), regardless of whether it is understood that a Smart City has other purposes beyond the economic development of the city. In this sense, Friedrich et al. (2021) describe a Smart City as a hybrid organization composed of competing institutional logics: the market logic versus the social welfare logic. In this sense thanks to the so-called smart computing technologies, an innovation habitat is generated (Entrepreneurship for Sustainable Development, 2018), enabling the exploration and exploitation of new opportunities and business models not exclusive limited to ICT sector (Kraus et al., 2015; Barba-Sánchez et al., 2019). Proof of this cross-sectorial impact has been evidenced by its resilience in times of economic crisis (Paroutis et al., 2014).

A Smart City combines an attractive location for the development of emerging industries and entrepreneurship, thanks to potential inter-firm linkages (Richter et al., 2015; Snieska et al., 2019), with smart development, thanks largely to network effects in the adoption of technologies (Lee et al., 2014). Ultimately, in the words of Hollands (2008), a Smart City can be understood as a high-tech variant of the entrepreneurial city. However, the success of this type of initiative is based on the attraction and retention of trained people, so the satisfaction of citizens with the infrastructure of these smart cities can be key to effectively increase the business activity. Based on this latter statement, we propose to include the following hypothesis into our model:

Hypothesis 4: Citizens’ satisfaction with the city’s infrastructure influences business activity.

The inclusion of the citizens’ satisfaction with the city’ infrastructures should allow us to examine the role of the ongoing Smart City initiatives, characterized by the penetration of IT into numerous city management tasks and activities.

## Information Technologies Education and Business Activity

According to Carnevale et al. (2011), STEM workers are the source of growth and innovation. However, one indicator of the importance of STEM skills is that there are not enough STEM-skilled workers to successfully contribute to the global-economy demands. In fact, from 2003 to 2013, the number of people working in occupations related to STEM grew by 12%, three times faster than the total employment rate in the EU. Occupations in these fields now account for 7% of all jobs and the demand for skills linked to these disciplines is anticipated to increase, particularly in the area of information and communications technology (ICT) (EU Stem Coalition, 2016).

As we stated in the introduction, Nelson and Phelps (1965) or Akhvlediani and Cieślik (2020) remark the importance of the role of education in the growth of economic activity as an accelerator of the process of technological diffusion and training of human capital. Nevertheless, a large part of success in education comes from teacher’s knowledge. In fact, although the STEM paradigm is closely related to technology, it is important to note that besides of counting with the IT infrastructure, its effective use in the teaching/learning processes is essential to get the most from the investments and efforts made by all actors (Tondeur et al., 2016). This return will heavily rely on the teaching staff training and involvement in integrating the use of IT technologies (Law et al., 2016; Tondeur et al., 2016).

However, the lack of technological knowledge on the part of teachers, which has been noted in the specialized literature (Kovarik et al., 2013; Chai et al., 2019), questions the validity of the concept of education as a diffuser of technology and a generator of business activity. Thus, it is observed that countries with fewer years of schooling and/or enrollment rates, which are common measures of the level of human capital training, sometimes show higher rates of economic growth than countries with higher aggregate educational levels (Akhvlediani and Cieślik, 2020). In this sense, authors such as Hanushek and Kimko (2000) point out that the most reliable educational level measures should focus on items related to schooling quality.

In this work, we address the development of a model to evaluate the impact of citizen priorities given to education on the generation of skilled workers and entrepreneurial activity. To this end, we propose the following hypothesis:

Hypothesis 5: The priority that citizens give to education moderates the relationship between the technological capacity of the school and business activity.

The inclusion into the model of the priority given by the citizens to the education, Hypothesis 5, as a variable that modulates the quality of the educational ecosystems should add light on the role played by all the elements of the school ecosystems.

As already mentioned, our study takes a starting point the data reported in the IMD Smart City Index (2019), and PISA reports. However, different from most up-to-date studies evaluating the secondary education programs’ outcomes, we explore the relationship between IT secondary education ecosystems and growth factors, mainly business activity, in a Smart City-based economy.

**FIGURE 1:**
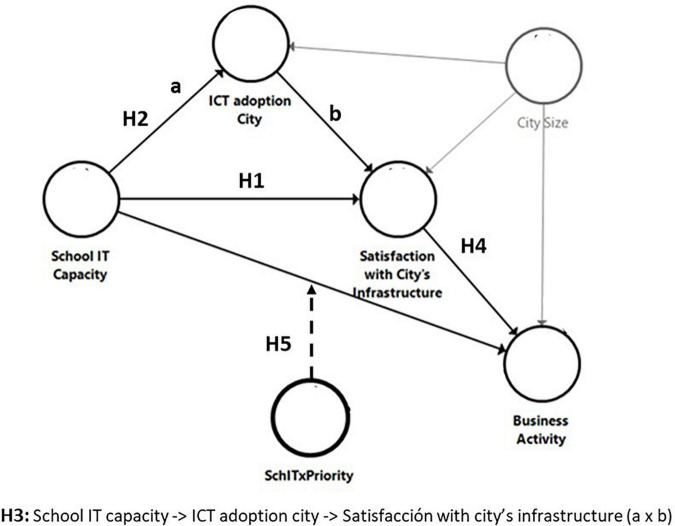
Proposed model.

To sum up, the proposed model is represented in Figure 1.

## Empirical Evaluation

### Data and Variables

In the framework of this research work, the following four sources of information have been used: the 2018 PISA, the IMD Smart City Index (2019), 2020 World Bank report (Doing Business, 2020), and United Nations Human Development Index (HDI, 2020). Different to most recent works making use of the PISA report and other sources of information (Hu et al., 2018; Xiao et al., 2019) on assessing the school performance of youngsters, the main goal of our study is to develop a model relating the efforts being made on developing and deploying IT resources and the business development of Smart City initiatives. According to Dolma (2010: 172, paragraph 1 on section 4), when the variables of the inquiry are operationalizations of the attributes of entities with different levels, there is no inconvenience in conceptually accepting that the higher level variables are an individual attribute of the lower level unit of analysis. Thus, both School IT Capacity and business activity are considered contextual attributes of the city.

Furthermore, since the focus of our research is on secondary education, we should note that the effect of secondary education on business activity will occur with a time lag (Simmons(ed.), 2016). This effect is the so-called “time lag dilemma.” Therefore, we have correlatively organized the years of the information sources: the oldest corresponding to years of school IT capacity followed by the ones relating to business activity. Accordingly, we have extracted from the various reports the following variables:

School IT Capacity: Data for this variable are retrieved for the well-known PISA report (PISA Data, 2018). In particular, we have chosen the following eleven items from the 2018 PISA database collected under the heading of School’s capacity using digital devices:

a. The number of digital devices connected to the Internet is sufficient
b. The school’s Internet bandwidth or speed is sufficient
c. The number of digital devices for instruction is sufficient
d. Digital devices […] are sufficiently powerful in terms of computing capacity
e. The availability of adequate software is sufficient
f. Teachers have the […] skills to integrate digital devices in instruction
g. Teachers have sufficient time to prepare lessons integrating digital devices
h. Effective professional resources for teachers to learn how to use digital […]
i. An effective online learning support platform is available
j. Teachers are provided with incentives to integrate digital devices in […]
k. The school has sufficient qualified technical assistant staff.

ICT adoption City: 18 items related to Technology pillar of a city from IMD-SUTD Smart City Index (SCI) 2018 (IMD Smart City Index, 2019) have been considered. This report ranks 102 cities worldwide by capturing the perceptions of 120 residents in each city. Technology pillar describing the technological provisions and services available to the inhabitants.

Satisfaction with City’s infrastructure: 18 items from SCI 2018 (IMD Smart City Index, 2019) with respect to the structures pillar referring to the existing infrastructures of the cities have been used.

School Priority: The data for this variable have been achieved from SCI 2018 (IMD Smart City Index, 2019). According to this report, “Priority Areas summarizes the areas that the respondents perceive as the priority area for their city. From a list of 15 indicators, survey respondents were asked to select 5 that they perceived as the most urgent for their city.” School Priority is one of the these15 indicator.

Business Activity: In order to get the information related to this variable 4 items have been taken into account:

a. New Business Density: This item considers new registrations per 1,000 people ages 15–64. Data have been collected from Doing Business (2020) report, excepting China and United States, which data were taken from www.statista.com.
b. The ease of doing business score measures the gap between a particular economy’s performance and the best practice and serves as basis for the ease of doing business rankings. The values for this variable has been obtained from Subnational Doing Business (2020) that considers 543 locations in 78 countries.
c. United Nations Human Development Index. The HDI was created to emphasize that people and their capabilities should be the ultimate criteria for assessing the development of a country, not economic growth alone. The values for this variable have been got from HDI (2020).
d. Gross National Income per capita expressed in US dollars for 2017 year. Those data have been achieved from IMD Smart City Index (2019).

Finally, the size of the city, as measured by the number of inhabitants according to the United Nations World’s Cities in 2018 report (United Nations Population Division, 2018), was used as a control variable, since it can affect both business activity and the decision to adopt ICT by those responsible for the city, as well as citizens’ perception of the adequacy of the city’s infrastructure.

To test the hypotheses, structural equation modeling (SEM) has been applied as it is especially recommended to test mediation hypotheses (Nitzl et al., 2016). Specifically, we used the partial least square (PLS) technique with the SmartPLS 3.2.9 statistical program (Ringle et al., 2015).

### Results

#### Assessment of the Measurement Models

Before contrasting the hypotheses proposed in the model, the measurement model must be evaluated by examining the individual reliability of the indicators of each construct, the reliability of the construct, the convergent validity, and the discriminant validity. To ensure the individual reliability of the indicators, they should all have loads equal to or greater than 0.707. However, Hair et al. (2017), point out that items whose loads are between 0.401 and 0.707 should also be retained, as long as this does not affect the quality of the measurement model, in order to preserve the content validity of the scales used. In our case, all loads are above the recommended values.

Table 1 shows the values of each construct with respect to the three measures of construct reliability, Cronbach’s Alpha and Dijkstrqa-Henseler’s rho_A, Composite reliability. For all constructs, the three measures exceed the minimum value of 0.7 and even the most advisable value of 0.8. With respect to convergent validity, measured by the Average Variance Extracted (AVE), all constructs exceed the minimum value of 0.5.

**TABLE 1:** Reliability estimates and convergent validity of the measurement model.

Construct	Cronbach’s Alpha	Dijkstrqa-Henseler’s rho_A	Composite reliability (CR)	Average variance extracted (AVE)
School IT capacity	0.969	0.979	0.974	0.774
ICT adoption city	0.980	0.982	0.981	0.747
Satisfaction with city’s infrastructure	0.969	0.974	0.972	0.665
Business activity	0.842	0.902	0.898	0.696

*All constructs are estimated in Mode A.*

As for the discriminant validity, Table 2 includes both the Fornell-Larcker criterion and the heterotrait-monotrait ratio (HTMT), which is more demanding, according to Henseler et al. (2016). In both cases, the constructs in our model meet the necessary conditions. In the first of them, the square root of the HTMT of a construct (in the main diagonal) is greater than its correlations with the rest of the constructs (lower part of the diagonal); and in the second of them, all the values of the HTMT are below 0.85 (upper part of the diagonal).

**TABLE 2:** Discriminant validity of the measurement model based on Fornell-Larcker and HTMT_0_._85_ criteria.

Construct	School IT capacity	ICT adoption city	Satisfaction with city’s infrastructure	Business activity
School IT capacity	**0.880**	*0.420*	*0.478*	*0.330*
ICT adoption city	0.410	**0.864**	*0.765*	*0.284*
Satisfaction with city’s infrastructure	0.471	0.758	**0.816**	*0.351*
Business activity	0.256	–0.084	0.310	**0.834**

*Diagonal elements (bold) are the square root of variance shared between the constructs and their measures (AVE).*

*Italic values above the diagonal elements are HTMT_0_._85_ values.*

*Values below the diagonal elements are the correlations between constructs.*

#### Assessment of the Structural Model

Once the measurement model has been evaluated, the structural model is evaluated by analyzing the collinearity between the constructors through the VIF values and the significance of the relationships between the constructors. In our model, all VIF values are well above the recommended value of 3 (Hair et al., 2019), being the highest of 1.467 between School IT Capacity and Business Activity.

To test the significance, a bootstrapping (10,000 resamples) based on percentile confidence intervals has been performed. As seen in Table 3, with respect to direct effects, the technological capacity of the school significantly influences the satisfaction of citizens with the infrastructure of the cities (H1: β = 0.134; *p* < 0.05), as well as the level of ICT adoption (β = 0.773; *p* < 0.001) and the size of the cities (β = −0.384; *p* < 0.001). It should be noted that the latter has a negative impact, i.e., the larger the size of the city, the lower the citizen satisfaction with its infrastructure becomes. This finding is a relevant result taking into account that the sample is made up of cities with a population between 374,000 and 38,001,000 inhabitants. Furthermore, the technological capacity of the school also significantly influences the level of ICT adoption in the cities (H2: β = 0.421; *p* < 0.001). In this case, the size of the city positively and significantly influences the level of ICT adoption in cities (β = 0.204; *p* < 0.05). Finally, the citizen satisfaction with the city’s infrastructure significantly influences the city’s business activity (H4: β = 0.227; *p* < 0.10). Our analysis also shows that priority that the citizens give to education and the city size also significantly influence business activity. In contrast, it is observed that the technological capacity of schools does not a significantly influence the city’s entrepreneurial activity. The latter outcome could have several possible explanations, e.g., a time lag that would be interesting to analyze in future research. Another possible explanation could be the existence of mediating variables, which have not been hypothesized in this paper. We should therefore explore this moderating effect at the end of this section.

**TABLE 3:** Effects on endogenous constructs.

Construct	Direct effect	*t-value*	*p-*value	Percentile confidence interval	Explained variance (*R*^2^)
**Satisfaction with city’s infrastructure (*R*^2^ = 0.745)**					
H1: School IT capacity	0.134	2.379	0.017	[0.019, 0.244]	0.063
ICT adoption city	0.773	15.321	0.000	[0.670, 0.872]	0.586
City size	−0.384	6.880	0.000	[–0.499, −0.281]	0.096
**ICT adoption city** (*R*^2^ = 0.210)					
H2: School IT capacity	0.421	5.222	0.000	[0.254, 0.566]	0.173
City size	0.204	2.555	0.011	[0.045, 0.360]	0.037
**Business activity** (*R*^2^ = 0.304)					
School IT capacity	0.102	0.808	0.419	[–0.126, 0.360]	0.026
H4: Satisfaction with city’s infrastructure	0.227	1.910	0.056	[–0.018, 0.446]	0.070
Education priority	0.324	3.782	0.000	[0.154, 0.487]	0.039
H5: School IT x priority	0.141	1.863	0.062	[–0.016, 0.281]	0.046
City size	−0.330	3.493	0.000	[–0.525, −0.148]	0.123

*Paths from hypothesis assessed by applying a two-tailed test at 5% of significance level [2.5%, 97.5%].*

*Bootstrapping based n = 10,000 bootstrap samples.*

With respect to the explanatory power of the model, the construct Citizen Satisfaction with the city’s infrastructure has an R2 of 0.745, so the model explains more than 67% of the variance of this construct, indicating substantial explanatory capacity, according to Chin (1998). Specifically, the level of ICT adoption by the city explains 58.6% of the endogenous variable, while the other two exogenous variables explain less than 10%. However, in the case of the constructs Level of ICT adoption by the city and Business activity, the explanatory capacity of the model is weak, since both have an R2 below 0.33, although they exceed the minimum required of 0.19.

Table 4 shows the results of the mediation of the ICT Adoption Level by the cities in the relationship between the Technological capacity of the schools and the Satisfaction of the citizens with the infrastructure of the city (H3: β = 0.460; *p* < 0.001). This mediation is partial, given that the direct effect is significant, and complementary, given that both the direct and indirect effects are positive (Nitzl et al., 2016). It should be noted that the size of the indirect effect, which is estimated through the Variance Accounted For (VAF) index, is much larger than the direct effect. Moreover, this partial mediation is an indication that there could be another additional mediating variable whose indirect effect also has the same direction as the direct effect.

**TABLE 4:** Summary of mediating effect tests.

Hypothesis	Total effect path (*p*-value)	Direct effect path (*p*-value)	Indirect effect		
			Path (*p*-value)	PCI	VAF (%)
H3: School IT capacity → ICT adoption city → Satisfaction with city’s infrastructure	0.460 (0.000)	0.134 (0.024)	0.326 (0.000)	[0.191, 0.449]	70.87
School IT capacity → Satisfaction with city’s infrastructure → Business activity	0.206 (0.048)	0.102 (0.419)	0.104 (0.070)	[–0.010, 0.216]	50.49

*PCI: Percentile Confidence Interval.*

*Paths from hypothesis assessed by applying a two-tailed test at 5% of significance level [2.5%, 97.5%].*

*Bootstrapping based n = 10,000 bootstrap samples.*

**FIGURE 2:**
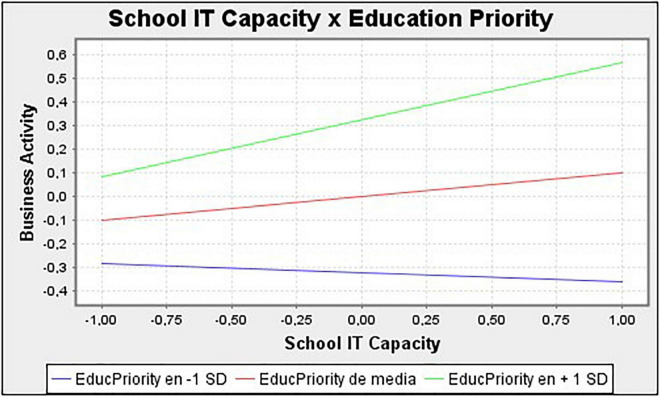
Simple slope graph of the moderating effect using PLS.

Finally, we study the relationship between Technological Capacity of Schools and Entrepreneurial Activity to answer the moderating effect of Education as a Citizen Priority (Hypothesis 5). In Figure 2, we observe that for higher levels of this variable, this effect increases based on the size of the interaction (0.102 + 0.141 = 0.243) and for lower levels, this effect decreases, even changing sign (0.102 − 0.141 = −0.039).

**FIGURE 3:**
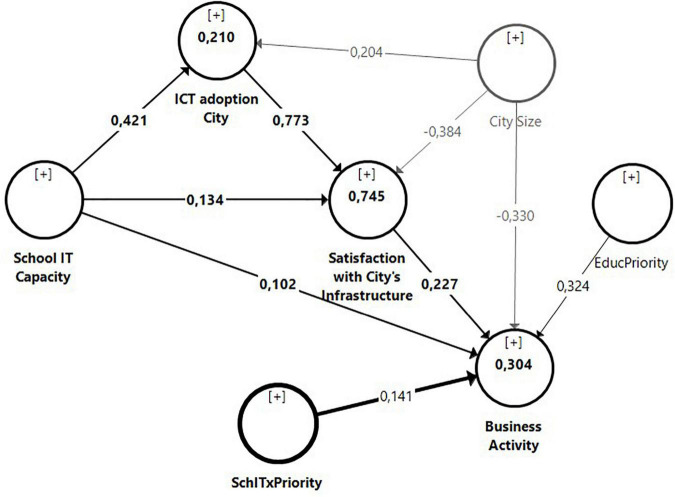
Final PLS estimated model.

In summary, the five hypotheses proposed are corroborated by the empirical data analyzed. The technological capacity of the school has a positive and significant influence on both the satisfaction of citizens with the city’s intra-structure (Hypothesis 1) and on the adoption of ICT by the city (Hypothesis 2). Moreover, the latter mediates the relationship between the first two (Hypothesis 3), so two relevant effects are observed: one direct and the other indirect through the mediating variable. Citizen satisfaction with the city’s infrastructure also has a positive and significant influence on the city’s business activity (Hypothesis 4). Finally, the priority that citizens give to education moderates the relationship between the technological capacity of the school and business activity (Hypothesis 5). The results of the estimated model are shown in Figure 3; specifically, the path coefficients (β) and the determination coefficients (*R*^2^).

## Main Findings and Discussion

The results reported in this study reveal the relationship between the technological capacity of the secondary-school capacity, and the business activity of a Smart City. Past studies have recognized the role of education as a disseminator of technology and generator of entrepreneurial activity in urban areas (Nelson and Phelps, 1965). However, the empirical evidence to date has been contradictory (Akhvlediani and Cieślik, 2020). In this sense, the novelty of this study is that the inclusion of the relevance of IT secondary education as a moderating variable of this relationship provides revealing information (Figure 2). The study shows the existence of a positive relationship between the IT capacity of secondary schools and the resulting entrepreneurial activity of the city. However, when the priority given to the IT capacity of schools is low or even negative, the effect over the entrepreneurial activity is negligible.

This result shows that in environments concerned with education, where there is a greater awareness of the vital role of education in socio-economic development, business activity is mainly based on the technological skills and knowledge that citizens have acquired. Furthermore, our model and results allow us to identify the shortcoming of some previous studies that have not revealed that significant investments in education should lead to more remarkable economic growth (Akhvlediani and Cieślik, 2020). Thus, the level of citizen awareness of the importance of education multiplies, positively or negatively, the effect of such investments in education. Therefore, our recommendations to education policymakers go in this direction. That is to say, social awareness of the value of education should dignify the role of the educational institution and teachers in parallel with the allocation of resources. Furthermore, promoting a vision of the school as an ecosystem where teachers and students interact by developing their skills and exploring their knowledge as sources of benefit would undoubtedly help citizens improve their perception of the usefulness or desirability of allocating resources to education.

On the other hand, it is observed that the intelligence of the city influences its business activity, in line with the results of Barba-Sánchez et al. (2019). The novelty of this study is that the intelligence of cities has been broken down into the two dimensions identified by the IMD Smart City Index (2019): technological, which measures the level of ICT adoption by cities, and structural, which reflects the level of services available in the city through citizen satisfaction with them. Our model indicates that the technological capacity of the city is influenced by the technological capacity of the school and directly affects the structural dimension of the city’s intelligence, which in turn contributes to its business activity. Although there are more and more studies that support the economic growth capacity of smart cities, their attractiveness for business location (Snieska et al., 2019) and even their capacity to generate sustainable competitive advantages (Strielkowski et al., 2020), our results show the complexity in the relationship between city intelligence and economic growth. Hence, the recommendation that follows has to do with the involvement of citizens in the definition and implementation of the concept of Smart City in a specific environment. It is not only a matter of implementing technology in all areas of the socio-economic life of the city, but also of understanding the needs of its citizens and satisfying them in a more efficient way. It is neither understandable nor efficient, in line with Zandbergen and Uitermark (2020), to have a Smart City without “smart citizens” who value the possibilities offered by such an intelligent environment and have the capacity to take advantage of them for their greater well-being and quality of life (Ugljanin et al., 2020). There is no doubt that the promotion of STEM studies in the field of secondary education can contribute to forming these “smart citizens,” capable of making their well-being compatible with the Sustainable Development Goals of the 2030 Agenda.

## Conclusion and Future Research

The main findings of this work have verified the benefits of investing and adapting the secondary school curricula on the development of smart city initiatives. Efforts to provide secondary schools with adequate IT capabilities by public-private institutions contribute to the adults of the future assuming more successfully and satisfactorily their responsibilities as workers and citizens of environments in which digital transformation and IT integration is an unstoppable fact, ratifying the first two hypotheses. Furthermore, it is confirmed that the IT endowment of the city mediates the relationship between the IT capacity of the school and the satisfaction of the cities with the IT infrastructure of their city, which is undoubtedly consistent with a policy that is committed to IT integration in all social areas in a stable manner over time. In other words, if adolescents are trained for tools that will not be provided to them in their adult life, they may feel frustrated in such an environment, corroborating hypothesis three.

On the other hand, it is empirically contrasted that the Smart City is a favorable environment for economic development and entrepreneurial activity (hypothesis 4), in the same sense that has been previously pointed out by the specialized literature (Sauer, 2012; Barba-Sánchez et al., 2019; Snieska et al., 2019). However, the novelty of this work is the ratification of the moderating role that the priority given to education in a given environment has on the relationship between the IT capacity of high schools and the entrepreneurial activity of that environment (hypothesis 5). In this sense, the practical recommendations to those responsible for educational policies go in the direction of promoting IT from an early age, not only as a way to improve future economic development through the better preparation of their professionals, but also to favor the integration of their citizens in all aspects of their city, reducing their sense of frustration with institutions.

However, further efforts should be carried out requiring the availability of more and wider variety of data sources. First, the unit object of this study is the city. However, this has not been the unit of measurement for all variables. While for the variables IT adoption city, satisfaction with city’s infrastructure, education priority and city size the data refers to the city itself. In the case of the other two statistics, school IT capacity and business activity, they are only available at the country level. Although this fact does not distort the results obtained, since these variables are considered as individual attributes of the cities, it is proposed for future research to test the model at the country level by performing Hierarchical Linear Modeling (HLM), as suggested by Dolma (2010). In addition, there is a debate in the reference literature about city limits, differentiating the concept of city proper, which identifies administrative boundaries, from that of metropolitan area, defined as the zone of economic and social influence of that city proper (United Nations Population Division, 2018). Authors such as Sassen (1991) or Andersen et al. (2011) are committed to extending the focus of interest to these metropolitan areas, given that city limits, understood in political-administrative terms, are diluted in socio-economic and cultural terms. In this line of our future research efforts, there are two main challenges: the consensual definition of the physical limits of metropolitan areas and the difficulty of obtaining specific data on them.

Second, the IMD-SUTD Smart City Index (SCI) 2018 (IMD Smart City Index, 2019) only collects information on medium and large cities, with a clear bias toward small cities. In particular, Geneva is the city with the smallest population with 371,000 inhabitants (United Nations Population Division, 2018). As we have already mentioned, the citizens of medium-sized cities do not perceive significant differences in the quality of the infrastructures deployed in their cities and those available in larger cities. However, they perceive some negative aspects derived from greater size (see Rérat, 2012). In this sense, it would be worth replicating this research in smaller cities (less than 300,000 people) to explore if the conclusion also apply to smaller communities.

Another factor worth of exploring will include additional mediating variables between school IT capacity, satisfaction with city’s infrastructure and business activity, such as the importance of introducing students into entrepreneurship. In this sense, in the report by the OECD (Organization for Economic Cooperation and Development), Lackéus (2015) pointed that “all students can and should train their ability and willingness to create value for other people. This is at the core of entrepreneurship and is also a competence that all citizens increasingly need to have in today’s society, regardless of career choice.” Thus, it would be interesting to investigate in future works the influence of entrepreneurial education on satisfaction with city’s infrastructure and business activity.

Furthermore, an issue that we have also not addressed in this research paper is the time lag in the effects of ICTs (a long-run perspective on ICTs) that Haider et al. (2021) have observed. According to these authors, the most notable effects of ICT incorporation in an organization are only observable with a considerable delay, even of several years, due to the long processes of ICT adoption and integration. In this sense, a longitudinal analysis of the effects of the integration of ICTs both in schools and in cities, in the Business activity could highlight the existence and relevance of such delays.

Finally, another interesting line of future research would be to examine in greater depth the impact of the priority that the population of a territory gives to education on its own economic development. In our case, we have measured this variable in a unidimensional way. Nevertheless, the complexity of gathering the perception of individuals advises the use of multidimensional scales that identify, for example, the priority educational areas with respect to STEM content or entrepreneurial education for quality education, in line with the definition made by the Sustainable Development Goals (SDG 4) (2030 Agenda, 2015).

## Data Availability Statement

Publicly available datasets were analyzed in this study. This data can be found here: https://www.oecd.org/pisa/data/, https://www.doingbusiness.org/en/reports/subnational-reports, https://www.imd.org/research-knowledge/reports/imd-smart-city-index-2019.

## Author Contributions

VB-S contributed to searching and processing of the data, statistical analysis, tables, and results. LO-B made intellectual contribution to the work and reviewed the final version of the manuscript. EA-A developed the literature review and wrote the manuscript. VB-S and EA-A developed the discussion and conclusion. LO-B and EA-A contributed to searching for references. All authors contributed to the article and approved the submitted version.

## Conflict of Interest

The authors declare that the research was conducted in the absence of any commercial or financial relationships that could be construed as a potential conflict of interest.

## Publisher’s Note

All claims expressed in this article are solely those of the authors and do not necessarily represent those of their affiliated organizations, or those of the publisher, the editors and the reviewers. Any product that may be evaluated in this article, or claim that may be made by its manufacturer, is not guaranteed or endorsed by the publisher.
